# Reprogrammed *Pteropus* Bat Stem Cells as A Model to Study Host-Pathogen Interaction during *Henipavirus* Infection

**DOI:** 10.3390/microorganisms9122567

**Published:** 2021-12-11

**Authors:** Noémie Aurine, Camille Baquerre, Maria Gaudino, Christian Jean, Claire Dumont, Sylvie Rival-Gervier, Clémence Kress, Branka Horvat, Bertrand Pain

**Affiliations:** 1 Stem Cell and Brain Research Institute, University of Lyon, Université Lyon 1, INSERM, INRAE, U1208, USC1361, 69500 Bron, France; noemie.aurine@inserm.fr (N.A.); camille.baquerre@inserm.fr (C.B.); christian.jean@inserm.fr (C.J.); sylvie.gervier@inserm.fr (S.R.-G.); clemence.kress@inserm.fr (C.K.); 2 International Center for Infectiology Research (CIRI), University of Lyon, Université Claude Bernard Lyon 1, INSERM, U1111, CNRS, UMR5308, ENS Lyon, 69007 Lyon, France; mariagaudino01@gmail.com (M.G.); dumont.claire3@gmail.com (C.D.)

**Keywords:** bats, stem cells, reprogramming, emerging infection, *Henipavirus*, Nipah virus, innate immunity, transcriptome, interferon-stimulated gene

## Abstract

Bats are natural hosts for numerous zoonotic viruses, including henipaviruses, which are highly pathogenic for humans, livestock, and other mammals but do not induce clinical disease in bats. *Pteropus* bats are identified as a reservoir of henipaviruses and the source of transmission of the infection to humans over the past 20 years. A better understanding of the molecular and cellular mechanisms allowing bats to control viral infections requires the development of relevant, stable, and permissive cellular experimental models. By applying a somatic reprogramming protocol to *Pteropus* bat primary cells, using a combination of ESRRB (Estrogen Related Receptor Beta), CDX2 (Caudal type Homeobox 2), and c-MYC (MYC proto-oncogene) transcription factors, we generated bat reprogrammed cells. These cells exhibit stem cell-like characteristics and neural stem cell molecular signature. In contrast to primary fibroblastic cells, these reprogrammed stem cells are highly permissive to henipaviruses and exhibit specific transcriptomic profiles with the particular expression of certain susceptibility factors such as interferon-stimulated genes (ISG), which may be related to viral infection. These *Pteropus* bat reprogrammed stem cells should represent an important experimental tool to decipher interactions during henipaviruses infection in *Pteropus* bats, facilitate isolation and production of bat-borne viruses, and to better understand the bat biology.

## 1. Introduction

The emergence of zoonotic diseases is increasing globally; thus the ability to predict and prevent viral epidemics is a major objective for public health organizations. Among all emerging infectious diseases, approximately 60% are of zoonotic origin [1] and bats are largely responsible for many of them as hosting a higher number of human and animal zoonotic viruses compared to other mammals [2,3]. Among others can be found Henipaviruses [4] (Nipah (NiV) and Hendra (HeV)), Filoviruses (Marburg [5] and Ebola viruses [6], Lyssaviruses [7], and coronaviruses including the Severe Acute Respiratory Syndrome (SARS-CoV) ([8], the Middle East Respiratory (MERS-CoV)) [9], and probably the SARS-CoV-2 [10]. The majority of these agents are among the most virulent pathogens emerging from animal reservoirs and are capable of infecting a broad range of species, including humans, while remaining asymptomatic in bats. Studying the bat immune system and bat/virus interactions provides valuable insight into the mechanisms underlying successful control of viral infection, and may lead to novel approaches to manage viral spillover and the development of new antiviral strategies for humans [11]. As infection studies in bats are difficult to implement due to the animal facility constraints and the absence of commercial bat suppliers, it is crucial to establish adequate research tools for comparative in vitro infection, for studying underlying mechanisms allowing the control of viral infection in bats, and for isolating new bat-borne viruses [12]. Availability of relevant cell lines is particularly important and represents a critical obstacle for further study of virus-host interactions [13].

Henipaviruses are negative-strand RNA viruses; both NiV and HeV are responsible for outbreaks of respiratory and neurological diseases in Southeast Asia and Australia, respectively, with a fatality rate of 40–100% [14]. The natural reservoirs of henipaviruses are bats belonging to the *Pteropus* genus [11,15]. Transmission of NiV from bats to humans occurs either through the infection of another animal, serving as a spillover host, including farm animals [16,17], or directly [18] via consumption of fruits or raw date palm juice contaminated with bat saliva or urine. The inter-human transmission was described in one-third of NiV outbreaks in Bangladesh [19]. In humans, the cellular targets of henipaviruses are endothelial, epithelial, and neural cells [20]. NiV and Henipa-like viruses have been detected molecularly and/or serologically in *Pteropus* bats from different Asian and African countries [21], and the worldwide distribution of these bat species poses a threat to future NiV pandemics [22]. 

Although several cell lines have been established from *Pteropus* bats, most of them present immortalized primary cells (PCs) [23,24,25]. Some of those cell lines present fibroblast-like morphology independently from the initial phenotype of PCs but other exhibits original phenotype such as the cuboidal lung and renal cell lines [23]. The requirement for using high multiplicities of infection (MOI) of 10 [25] or even 100 [23] to obtain a successful viral infection suggests low permissiveness of those cells for henipaviruses. The use of primary and immortalized cell lines limits our ability to isolate and study viruses that may show tropism for cell types that are more difficult to establish as primary cultures or to immortalize. In addition, although cell immortalization allows the generation of the continuously growing cell lines, useful for certain virology analyses, the initial primary phenotype is often lost following immortalization, modifying the interactions between virus and cell host and limiting thus drastically their utility [26] thereby emphasizing the need to develop more appropriate bat cells for further studies. 

Here, we generated *Pteropus* bat reprogrammed stem cells (bRSCs) from bat PCs (bPCs) using a combination of three transcription factors: ESRRB, CDX2, and c-MYC, previously shown to play a role in the maintenance and physiology of stem cells. The ESRRB (Estrogen related receptor beta) is a transcription factor involved in the maintenance of self-renewal and pluripotency embryonic stem cells and the efficient reprogramming to naïve pluripotency in the mouse model [27]. The CDX2 is a transcription factor involved in embryonic and intestinal development and also in trophoblast stem cells [28]. The c-MYC gene is a proto-oncogene encoding a nuclear phosphoprotein that plays a role in cell proliferation and is one of the main actors of the initial canonical somatic reprogramming gene combination [29]. These bRSCs exhibit typical pluripotent stem cells (PSCs) morphology, are recognized by SSEA-1 and EMA-1 stem-cell-specific antibodies, display PSCs-specific chromatin features and express a set of neural stem cell-associated genes. These bRSCs are much more susceptible to virus infection than the parental bPCs, providing a new model to investigate host-pathogen interactions. Interestingly, bRSCs, compared to bPCs, show a unique transcriptomic profile for genes that can be responsible for viral restriction and modulate susceptibility factors implicated in a viral cycle, interferon response, inflammatory pathway, or apoptosis. This novel cellular model will present an important experimental tool to contribute to a better understanding of the unique properties of bat-virus interactions and will help to decipher novel aspects of bat biology. 

## 2. Materials and Methods

### 2.1. Pteropus Bat Primary Cell Culture

Cell cultures of *Pteropus* bat flying fox (*P. giganteus*, also known as *P. medius*) and *P. vampyrus* (Yinpterochiroptera suborder) were generated from samples collected in Tiergarten Schönbrunn (Vienna, Austria), as described previously [30]. Briefly, wing-membrane skin biopsies were obtained during the regular veterinary checkup in accordance with national guidelines to minimize stress. In addition, lung and trachea samples were obtained from a female specimen found naturally dead and were not euthanized for the purpose os sample collections. Samples were washed with sterile PBS and transferred to Cryo-SFM freezing medium (PromoCell Bioscience Alive GMBH, Heidelberg, Germany, Cat# C-29910) on dry ice for shipment from the zoo. To obtain bat primary cell cultures (bPCs), samples were dissected in a Petri dish and explants derived from the trachea (PTCs), lung (PLCs), and alary membrane (PACs) were cultured at 37 °C/7% CO_2_ in gelatin-coated wells containing either fibroblast medium (FM) (DMEM/F12 supplemented with 10% fetal bovine serum (FBS), 2 mM L-glutamine, 1000 U/mL penicillin, 1000 U/mL streptomycin) or ESM1 medium (DMEM/F12 supplemented with 10% FBS, 2 mM L-glutamine, 1000 U/mL penicillin, 1000 U/mL streptomycin, 1% non-essential amino acids (100X), 1 mM sodium pyruvate, 0.1 mM β-mercaptoethanol, 1 ng/mL IL-6, 1 ng/mL IL-6 receptor, 1 ng/mL Mouse Stem Cell Factor, 5 ng/mL insulin-like growth factor-1, and 1000 U/mL leukemia inhibitory factor); this culture protocol is used routinely for avian stem cells [26]. References to all reagents used for cell culture are listed in Supplementary Table S1.

### 2.2. Reprogramming Vectors

CDNAs encoding human POU5F1, SOX2, KLF4, NANOG, c-MYC, and CDX2, and mouse ESRRB were cloned, sequenced, and inserted into an inducible pPB transposon backbone. All constructs were generated using the NEBuilder® HiFi DNA Assembly Master Mix system (New England BioLabs, Evry, France, Cat# E2621) and sequenced to validate correct cDNA insertion. Viral stocks of the Sendai viral vector expressing the POU5F1/OCT4, SOX2, KLF4, and c-MYC human genes, were purchased from CytoTune2.0 kit (Invitrogen, Thermo Fisher Scientific, Illkirch, France, Cat# A16517).

### 2.3. Generation of Reprogrammed Pteropus Bat Cells

bPCs were seeded in a 6-well plate and infected with Sendaï viruses containing KLF4-OCT4-SOX2 and c-MYC (5 PFU/cell), and with a virus containing KLF4 (3 PFU/cell), according to the protocol provided by the virus supplier (CytoTune2.0 kit, Invitrogen, Thermo Fisher Scientific, Illkirch, France, Cat# A16517).Cells were passaged 5 days after infection and seeded in two 55-cm^2^ flasks. The cells were cultured for 6 days in ESM1 or EPI medium (the medium was changed every 2 days). The EPI medium is serum-free and includes 50% DMEM/F12 and 50% Neurobasal medium, supplemented with B-27 Supplement, N-2 Supplement, 2 mM L-Glutamine, 1000 U/mL penicillin, 1000 U/mL streptomycin, 1 mM β-mercaptoethanol, 5 ng/mL basic-Fibroblast Growth Factor (b-FGF), and 10 ng/mL human Activin A. In a second approach, bPCs were modified by electroporation with different combinations of inducible transposons encoding OCT4, SOX2, KLF4, c-MYC, NANOG, ESRBB, and CDX2. bPCs cells were dissociated and centrifuged at 1200 rpm (300× *g*) for 5 min at ambient temperature. The cell pellet was washed in PBS, centrifuged again, and 1.2 × 10^6^ cells were recovered in 120 μL of R resuspension buffer of the Neon system (Neon, Life Technologies, Thermo Fisher Scientific, Illkirch, France, (Cat# MPK5000) containing 2 μg of pCAGPBase transposase-expressing vector and 4 μg of the reprogramming gene cocktail (31, 57). 100 μL of this cell-plasmid solution was electroporated (1500 V, 30 ms, 1 pulse) in a 100 μL tip (Neon, Life Technologies, Thermo Fisher Scientific, Illkirch, France Cat# MPK10096). After electroporation, cells were cultured in 3 mL of FM medium in a 6-well plate. Medium from electroporated cells was replaced after 24 h and cells were selected using 5 μg/mL of puromycin and 200 μg/mL of neomycin, depending on the resistance genes carried by the plasmids present in the mixture. Medium containing selection reagents was changed every 2 days for at least 1 week. At the end of the selection process (between 8 and 15 days), the cells were dissociated by 0.05% trypsin-EDTA (Life) and 2 × 10^5^ cells were seeded into a well in a 6-well plate containing 3 mL of ESM1, EPI, or ESM2 medium supplemented with 2 µg/mL of doxycycline. ESM2 medium includes DMEM/F12 supplemented with 10% FBS, 2 mM L-glutamine, 1000 U/mL penicillin, 1000 U/ mL streptomycin, 1% non-essential amino acids (100×), 1 mM sodium pyruvate, 0.1 mM β-mercaptoethanol, 5 ng/mL b-FGF, and 10 ng/mL human Activin A. References for all reagents used for cell culture are listed in Supplementary Table S1.

### 2.4. Characterization of Pteropus Bat Reprogrammed Cells

Cells were examined by electron microscopy and exponentially growing cells were subjected to cell cycle analysis as previously described [31,32]. Reactivity of cells with antibodies specific for SSEA-1, SSEA-3, SSEA-4, or EMA-1 as pluripotency-associated markers were tested as previously described [30]. The nuclear distribution of histone methylation marks (H3K9me3 and H3K27me3, as marks for constitutive and facultative heterochromatin respectively) was analyzed by immunofluorescence microscopy as described previously [33]. FastQ files were generated by RNAseq sequencing (Eurofins Genomics; Nantes, France, https://www.eurofinsgenomics.eu/ (accessed on 1 October 2021)) of bat PTCs as a starting material for reprogramming, along with two reprogrammed cell populations, E2 and ES2, which were established by culture, respectively, in EPI and ESM2 medium. For analysis, a reference index was created based on the *Pteropus* giganteus annotation GCF_902729225.1 from the NCBI database, which was assembled on April 15, 2020. FastQ files were pre-processed using TAGADA (https://github.com/FAANG/analysis-TAGADA, 12 October 2021, Genotoul plateform, Auzeville, France ) and the results were visualized with DRAMAA software (https://gitlab.com/hssgenomics/Shiny (accessed on 1 October 2021), version 1.4.0). Raw data and gene tables are available through the GSE134585 datasets (Supplementary Table S2). GO annotations were performed (Gene Ontology, http://geneontology.org/, current release 16 November 2021), using the human Gene symbols as references. An ISGs table [34] was crossed with (ECM_E2 + ECM_ES2)/2-vs-PTC DEGs (Supplementary Table S3), with an adjusted FDR value lower <0.05 using common gene symbols to generate an ISG volcano plot.

### 2.5. Pseudotyped Virus Infection Assay

Henipavirus pseudotyped particles were generated from rVSV-ΔG-RFP, a recombinant VSV in which the G protein envelope has been replaced with RFP (red fluorescent protein), as previously described [30]. Briefly, the attachment glycoprotein and fusion proteins of NiV/Malaysia, NiV/Bangladesh, NiV/Cambodia, and HeV were cloned from RNA isolated from each virus and inserted into a pCAGGS plasmid vector. For each viral isolate, plasmid vectors coding the glycoproteins were transfected into BSR-T7 cells using the TransIT®-LT1 Transfection Reagent (Mirus Bio, Souffelweyersheim, France, Cat# MIR 2300). 16 h post-transfection, cells were infected with rVSV-ΔG-RFP at an MOI of 0.3 to produce pseudotype VSVs for each isolate. The supernatant was collected 24 h post-infection and concentrated by ultracentrifugation (28,000 rpm for 2 h at 4 °C). Titration of viral stocks was done on Vero cells. Pseudotyped viruses were named after the virus providing the surface glycoprotein. To evaluate viral entry into different bat cells, cells cultured in 24-well plates (80% confluent and adherent) were infected at an MOI of 0.01 for 1 h at 37 °C. Then, the virus-containing medium was removed and cells were washed once with PBS. Finally, a fresh medium was added to cells for 6 h at 37 °C. The percentage of infected cells was assessed by measuring RFP fluorescence by flow cytometry (BD LSR Fortessa, BD Biosciences, Le Pont de Claix, France). 

### 2.6. Viruses

NiV-Malaysia isolate UMMC1; GenBank AY029767, (NiV-M), NiV-Bangladesh isolate SPB200401066, GenBank AY988601, (NiV-B), recombinant NiV-enhanced green fluorescent protein (EGFP) (rNiV-M-eGFP) [35] and NiV Cambodia isolate NiV/KHM/CSUR381, GenBank MK801755, isolated from P. lylei bat urine in Cambodia [26] (NiV-C) were produced and titrated on Vero E6 cells at the INSERM Jean Mérieux Biosafety Level 4 (BSL-4) laboratory in Lyon, France, with the highest level of biocontainment. Vero E6 cell line (ATCC#CRL-1586) obtained from the kidney of African Green Monkey and cells of the human carcinoma HeLa cell line (Cat# ATCC CCL-2) were cultured in DMEM medium supplemented with GlutaMAX (Life TechnologiesTM, Thermo Fisher Scientific, Illkirch, France, Cat# 61965-026) and 10% of heat-inactivated (30 min at 56 °C) fetal bovine serum (FCS) (Dutscher, Bernolsheim, France, Cat# S1810-500) and 15 μg/mL Gentamicin (Life TechnologiesTM, Thermo Fisher Scientific, Illkirch, France, Cat# 15750-037).

### 2.7. Henipavirus Infections of Bpcs

For NiV-M, NiV-B, and rNiV-M-eGFP, bPCs were plated in 6-well plates and upon reaching 80% of confluence were infected for 1 h at 37 °C at an MOI of 3, the highest tested MOI among those we had tested initially (MOI of 3, of 0.3 and 0.03) for the infection of bPCs using the highest biocontainment environment (BSL4). Next, the virus-containing medium was removed, and cells were washed once with PBS. Finally, a fresh medium was added to cells, followed by incubation at 37 °C for 24 h. Cells infected with NiV-M and NiV-B were fixed for 20 min in 4% formaldehyde solution. Cells were then permeabilized with PBS with 0.1% Triton X-100 and 3% normal horse serum (IF solution) for 20 min and then incubated with a primary rabbit anti-NiV N purified antibody (ValBex, Lyon, France) diluted at 1:1000 in IF solution 4 h at room temperature. After 3 washes, cells were incubated with AlexaFluor 555 donkey anti-rabbit secondary antibody (Invitrogen, Thermo Fisher Scientific, Illkirch, France, Cat# A31572) at dilution 1:750 in IF solution. DNA counterstaining with DAPI was done overnight at 4 °C. On the following day, cells were washed 3 times with IF solution, and fluorescent images were taken on a Zeiss Axiovert 100 M microscope (Carl Zeiss SAS—Microscopy, Marly le Roy, France). Cells infected with rNiV-M-eGFP were fixed for 20 min in 4% formaldehyde solution and the percentage of infected cells was assessed by measuring GFP fluorescence by flow cytometry (Navios, DS-14644A, BD Biosciences, Le Pont de Claix, France).

### 2.8. Henipavirus Infections of bPCs and bRSCs 

For NiV-M, NiV-B, and NiV-C, bPCs, and bRSCs were plated in 12-well plates and upon reaching 80% of confluence were infected for 1 h at 37 °C with the virus at an MOI of 0.1 (as the infection was saturating at an MOI of 1) using the highest biocontainment environment (BSL4). Next, the virus-containing medium was removed and cells were washed once with PBS. Finally, fresh medium was added to cells, followed by incubation at 37 °C for 0, 6, 16, 24, or 48 h. Cell morphology was observed under a Zeiss Axiovert 100 M microscope and photos were taken at 24 h post-infection using ImageJ software. For each time point, infected cell lysate was prepared using RLT buffer (Qiagen, Les Ulis, France, Cat# 79216) prior to RT-qPCR analysis according to a validated BSL-4 procedure. The supernatant was collected and kept at −80 °C until titration in a plaque assay on Vero E6 cells. Infections and titrations were performed in a BSL-4 facility at Jean Mérieux (Lyon, France). At the indicated time points, lysates prepared from infected cells were collected and RNA extracted using the NucleoSpin RNA kit (Macherey-Nagel, Hoerdt, France, Cat# 740955). The yield and purity of extracted RNA were assessed using a spectrophotometer (DS-11 FX; Denovix, Wilmington, DL, USA). Extracted mRNA was reverse-transcribed using SuperScript™ III Reverse Transcriptase (Invitrogen, Thermo Fisher Scientific, Illkirch, France, Cat# 18080). Real-time PCR was performed using Platinum SYBR Green qPCR SuperMix-UDG kit with ROX reference dye (Thermo Fisher Scientific, Illkirch, France, Cat# 11733038) and the StepOne plus PCR system (Applied Biosystems, Thermo Fisher Scientific, Illkirch, France). Data were analyzed using StepOne v.2.3 software (Applied Biosystems, Thermo Fisher Scientific, Illkirch, France,) and calculations were done using the 2ΔΔCT method. Expression was normalized to that of glyceraldehyde 3-phosphate dehydrogenase (GAPDH), as described previously [36]. The primers used are pGAPDH forward: ATCATCCCTGCTTCTACT and reverse: AGGTCAGATCCACAACT; and NiV-N forward: GGCAGGATTCTTCGCAACCATC and reverse: GGCTCTTGGGCCAATTTCTCTG. 

## 3. Results

### 3.1. Pteropus Bat Primary Cells (Bpcs) Exhibit Fibroblast-Like Morphologies and Limited Proliferation

**Figure 1:**
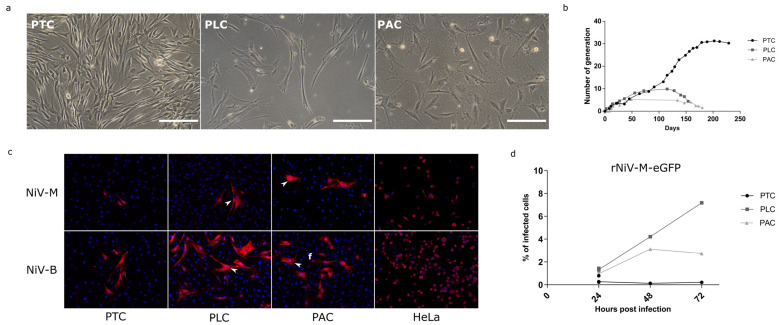
In vitro culture and Nipah viruses infections of bat primary cells (bPCs). (**a**) bPCs derived from the trachea (PTC), lung (PLC), and alary membrane (PAC) were observed under a light microscope. (**b**) Comparative growth curves of bPCs. (**c**) bPCs and HeLa cell line, used as control of infection, were infected with NiV-M and NiV-B isolates at an MOI of 3. At 24 h post-infection, the infected cells were visualized by NiV nucleoprotein immunostaining (red). DNA was counterstained with DAPI (blue). (**d**) bPCs were infected with the recombinant NiV-M virus expressing eGFP protein: rNiV-M-eGFP at an MOI of 3. The percentage of infected cells was quantified at 24, 48, and 72 h post-infection measuring GFP by flow cytometry.

A sampling of organs from *Pteropus* bats was carried out to generate in vitro models to study henipaviruses in their natural reservoir. Since the respiratory system is the entry route for henipaviruses in susceptible species, the trachea and lungs were sampled during a necropsy. In addition, alary membrane skin bio punches, which could be easily sampled, were harvested from several individuals. After mechanical dissociation, explants derived from the trachea (PTC), lung (PLC), and alary membrane skin (PAC) were cultured in gelatin-coated wells in different cell culture media: epithelial, stem cell, or fibroblast medium. Proliferative cells emerged from PTC, PLC, and PAC explants at 5, 15, and 30 days after plating, respectively, in fibroblast medium (FM) or ES-derived medium (ESM1). All cells exhibited a fibroblast morphology, with an elongated and flattened shape (Figure 1a). The growth curves of the bPCs were established, and we observed that PACs entered senescence after 4 to 5 generations, while PLCs became senescent after 10–11 generations. By contrast, PTCs continued to proliferate for more than 30 generations (200 days of continuous culture), and then they slowly and progressively but irreversibly stopped proliferating (Figure 1b). Thus, explant cultures allowed to generate bPCs from trachea, lung, and alary membrane with fibroblast-like morphology and a limited life span in vitro.

### 3.2. bPCs Present a Limited Permissivity to Henipavirus Despite High MOI

We then investigated whether generated bPCs were permissive to henipaviruses infection. bPCs were infected with two human isolates of Nipah virus: Nipah Malaysia (NiV-M) and Nipah Bangladesh (NiV-B) at a high MOI of 3. 24 h post-infection, few foci of infected cells were detected in the three types of bPCs while all human Hela cells, used as a positive control, were infected (Figure 1c). Syncytia were observed in PLC and PAC cultures (arrowheads). The number of stained foci was higher in cultures infected with NiV-B regardless of the primary bat cell type, suggesting that NiV-B is more infectious or less cytolytic than NiV-M in these bPCs. Quantification of the infection level was performed with a GFP expressing recombinant NiV-M (rNiV-M-eGFP). bPCs were infected with rNiV-M-eGFP at a high MOI and the GFP was measured post-infection by flow cytometry (Figure 1d). In all bPCs, less than 2% of cells were GFP positive 24 h post-infection. The percentage of infected cells increased during kinetic only in PLC, and reached 7% of infected cells 72 h post-infection. Thus, bPCs can be infected by both NiV-M and NiV-B although at a very low level.

### 3.3. Reprogramming of bPCs

To obtain a relevant model for henipaviruses infection studies, we aimed to generate cell types susceptible to *Henipavirus* infection and be capable of long-term proliferation, without using immortalizing agents. To overcome the limited lifespan of bat primary cells, we proceeded with the generation of *Pteropus* bat cells with stem cell features, including self-renewal and differentiation abilities. PTC was selected for the reprogramming approach, as they presented the longest proliferation capacity in culture (Figure 1b) and they are referred to as ‘bPCs’ in the rest of the manuscript. We initially compared the sequences of pluripotency genes between *Pteropus* bat and humans. Alignment of protein sequences derived from *P. vampyrus* and human genes revealed identities ranging from 87–99% between seven major pluripotency human genes, with the noticeable exception of NANOG, which showed only 70% identity (Supplementary Table S4). Due to the significant identity, we used the human genes for reprogramming. Next, we introduced the well-defined human OSKM gene combination into bPCs through Sendai viruses commercially available as detailed in methods in the presence or absence of hNANOG, which was expressed through a doxycycline-inducible transposon. Although the genes were delivered and expressed efficiently (Supplementary Figure S1b), long-term morphological changes were not observed up to 100 days post-infection (Supplementary Figure S1a). Delivering the same gene combination using inducible transposon vectors did not provide any better result, although an increase in cellular proliferation was observed, along with transient and partial morphological changes (Supplementary Figure S1c,d).

**Figure 2:**
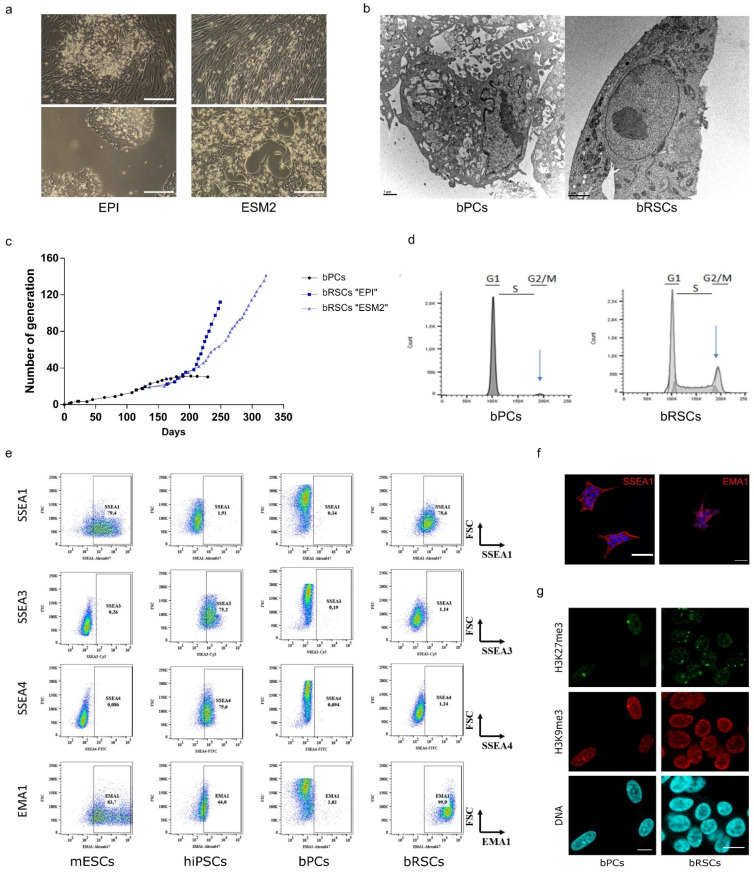
Generation and characterization of bat reprogrammed stem cells (bRSCs). bPCs were reprogrammed by inducing the expression of ESRRB, CDX2, and c-MYC, genes, and culture in either EpiStem (EPI) or ESM2 medium. (**a**) Morphology of bRSCs observed under a light microscope after 25 or 35 (top) and 170 (bottom) days of culture in EPI or ESM2 medium respectively. Scale bar, 200 μm. (**b**) Ultrastructural analysis of bRSCs by electron microscopy. Scale bar, 1 μm. (**c**) Comparative growth curves of bPCs and bRSCs. (**d**) Cell cycle analysis using propidium iodide staining of bPCs and bRSCs. (**e**) Flow cytometric analysis of bPCs and bRSCs for pluripotency markers compared to murine embryonic stem cells (mESCs) and human pluripotent stem cells (hiPSCs). (**f**) Immunostaining of pluripotency markers SSEA1 and EMA1 in bRSCs. (**g**) Histone post-translational modifications in bRSCs nuclei: immunodetection of H3K27me3 and H3K9me3, and DNA counterstaining with TO-PRO-3. Scale bar, 10 µm. Results labeled “bRSCs” correspond to bRSCs “EPI”. Similar results were obtained with bRSCs “ESM2”.

To find out the gene combination, which is efficient in bat cells, we tested a combination of different known transcription factors known to be expressed in early murine embryos and identified the combination of ESRRB, CDX2, and c-MYC (ECM) genes. This new combination delivered through doxycycline-inducible transposons was efficient on human, bovine, and equine primary cells to get drastic morphological changes and establishment of reprogrammed stem cells (cf infra 6. Patents). Transduced cells were cultured in two different cell-culture media suitable for stem cells: serum-free EPI medium or serum-containing ESM2 medium. In the EPI medium, foci showing typical stem cell morphology appeared at around 25 days after the addition of doxycycline; a longer period (35 days) was required for cells cultured in ESM2 medium (Figure 2a). The stable proliferation of bat reprogrammed stem cells (bRSCs) has continued for more than 110–140 generations, spanning 250–320 days (Figure 2c). The average doubling time in EPI medium was estimated to be around 24 h, and that in ESM2 medium around 35 h. Thus, the ECM combination expressed in bPCs allows the generation of new stem cell phenotypes with stable and constant proliferation rates.

### 3.4. bRSCs Show Stem Cell-like Characteristics

bRSC were shown to exhibit stem cell-like characteristics. bRSCs grew in compact colonies in both EPI and ESM medium, with morphology typical of pluripotent stem cells (PSCs): small and round, with a large nucleus, a high nucleo-cytoplasm ratio, and a prominent nucleolus (Figure 2a,b). Electron microscopy confirmed that the morphology and ultrastructure of bRSCs were very different from that of bPCs (Figure 2b). In particular, chromatin was distributed homogeneously throughout the nucleoplasm, without large zones of electron-dense heterochromatin that are typical of differentiated cells, and regularly observed in the nuclei of bPCs. The endoplasmic reticulum in bRSCs was abundant, but not dilated as in bPCs. In contrast to PCs, the bRSCs exhibit a long-term proliferation in both Epi and ESM2 media (Figure 2c). Cell cycle analysis of bRSCs revealed a PSCs-like profile with a short G2/M phase and a long S phase, unlike bPCs (Figure 2d). Expression of PSCs-specific antigens, including stage-specific embryonic antigens (SSEA-1, SSEA-3, and SSEA-4) and an epithelial membrane antigen (EMA-1) [37,38] was analyzed by immunostaining and flow cytometry (Figure 2e,f). Similar to murine embryonic stem cells (mESCs), but in contrast to human-induced pluripotent stem cells (hiPSCs), bRSCs were positive for SSEA-1 and EMA-1 and negative for SSEA-3 and SSEA-4. The nuclear distribution of some epigenetic marks in bRSCs was similar to the pattern observed in several PSCs types (Figure 2g). Indeed, the facultative heterochromatin marker H3K27me3 in bRSCs was distributed within several large foci, rather than being concentrated mainly at the inactivated X chromosome as in bPCs. Previously, such numerous prominent H3K27me3 foci were observed in both naïve mouse PSCs and pluripotent and reprogrammed avian cells [39]. Taken together, these different expression profiles strongly suggest that the following reprogramming, bRSCs share key features with PSCs from other species.

### 3.5. bRSCs Have a Specific Neural Stem Cell Molecular Signature

**Figure 3:**
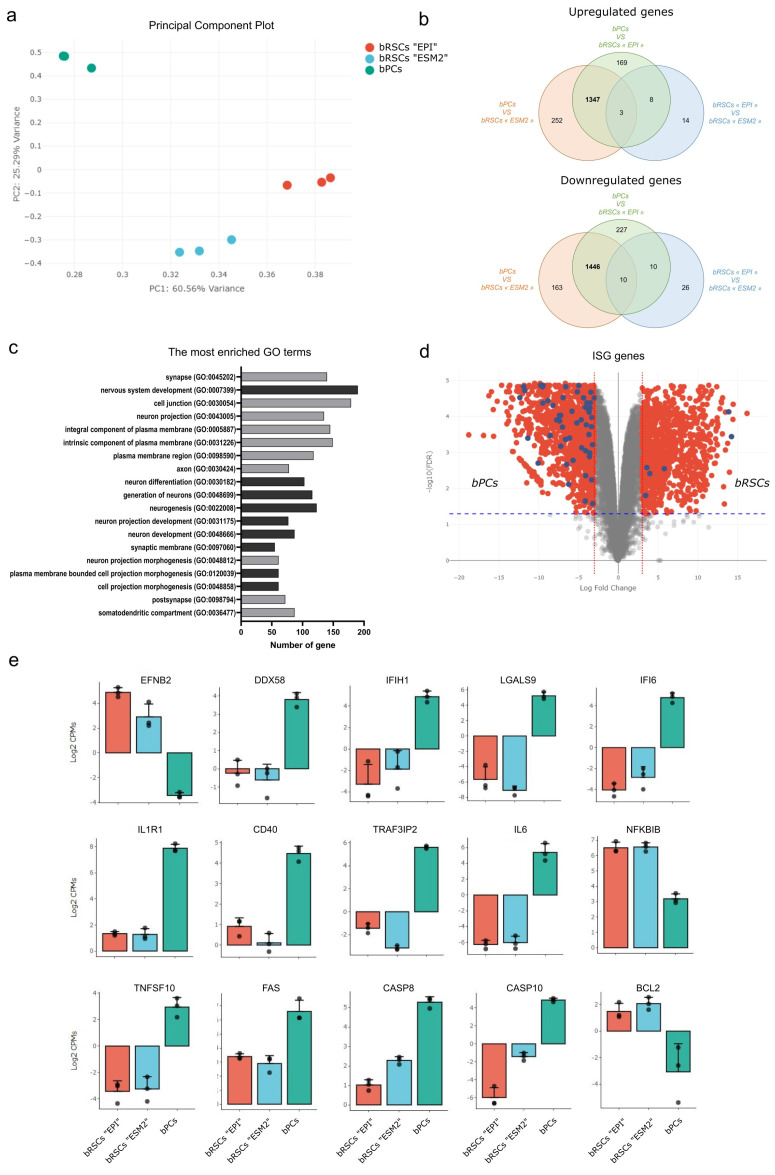
Molecular features of bat reprogrammed stem cells (bRSCs). (**a**) Principal component analysis (PCA) of RNA-seq results for bPCs and bRSCs. Principal Component 1 (PC1, x-axis) represents 60.56% and PC2 (y-axis) represents 25.29% of the total variation in the data. (**b**) Venn diagram of differentially expressed genes (DEGs) between bPCs and bRSCs cultured in “EPI” or “ESM2” medium. The numbers of common DEGs between the two bRSCs cultures are in bold. (**c**) Gene ontology (GO) term enrichment analysis of DEGs in bRSCs. The terms with the best FDR value were selected. Dark gray represents the GO biological process and light gray represents the GO cellular component. (**d**) Volcano plot representation of DEGs in bPCs versus bRSCs comparison. Red dots represent all DEGs with fold change >3 and blue dots represent DEGs corresponding to interferon-stimulated genes (ISGs). (**e**) Bar graphs of the relative expression levels of selected genes between bPCs and bRSCs in “EPI” and “ESM2” medium: EFNB2, DDX58, IFIH1, LGALS9, IFI6, IL1R1, CD40, TRAF3IP2, IL6, NFKBIB, TNFSF10, FAS, CASP8, CASP10, and BCL2.

To define the full transcriptome landscape of those bRSCs, we performed deep RNA sequencing of both bPCs and bRSCs RNA. Principal component analysis (PCA) of expressed genes indicated that bRSCs cultured in either EPI or ESM2 medium had expression profiles clearly distinct from those of bPCs (Figure 3a). More precisely, 2806 genes were differentially expressed, 1456 upregulated and 1350 downregulated, between bPCs and bRSCs, with a log-fold change in absolute value higher than 2 (LFC > 2) and a *p*-value > 0.05 (Figure 3b, Supplementary Table S2). If bRSCs do not express both POU5F1/OCT4 and NANOG key pluripotent genes, they are still positive for SOX2 and ZSCAN4, which play roles in telomere maintenance and long-term-genomic stability in embryonic stem cells [38], as they are positive for the transduced ECM genes. Gene ontology GO term enrichment analysis on 828 differentially expressed genes (DEGs) with an LFC > 3 between bPCs and bRSCs was done and the terms were classified according to the best FDR value. Interestingly, among the first 20 GO terms, 14 are associated with neural pathways as “nervous system development” (GO:0007399). In addition, 5 genes are associated with cell projections as “cell projection morphogenesis” (GO:0048858), 3 to the plasma membrane as “integral component of plasma membrane” (GO:0005887; and 1 to “cell junction” (GO:0043005). These expression profiles suggest that the reprogramming process with the ECM combination gave rise to a new neural-related stem cell type that differs from the initial bPCs. 

### 3.6. Bat Reprogrammed Stem Cells (Bat RSC) Express Henipavirus Entry Receptor and Present a Specific Intrinsic ISG Expression

We then looked for some viral susceptibility or restriction factors that can be highlighted by transcriptome analysis. The first was the entry receptor of the viruses. Henipaviruses use Ephrin B2 (EFNB2) and Ephrin B3 (EFNB3) molecules as cellular entry receptors in numerous species [40,41]. EFNB2 was among the main DEGs with an FC = 7.8 and was expressed at much higher levels in bRSCs than in bPCs (Figure 3e). However, no EFNB3 reads were detected in deep sequencing of RNA in both bPCs or bRSCs. The expression profile of interferon-stimulated gene (ISGs), known to modulate innate immune response during viral infection, was investigated. Genes defined as a mouse or human ISGs are represented in the volcano plot in blue among DEGs with LFC > 3 and *p*-value > 0.05. Interestingly, 57 ISGs were downregulated in bRSCs compared to bPCs while only 6 were upregulated (Figure 3d, Supplementary Table S3). Among these ISGs, DDX58 coding for RIG-I protein and IFIH1 coding for MDA5 protein, used as viral recognition receptor, are downregulated in bRSCs in comparison with bPCs (Figure 1e). In contrast to previously published constitutive expression of IFN-I in cells from *P. alecto* bats [42] neither type I nor type III interferons were differentially expressed between bPCs and bRSCs. Moreover, we have compared DEGs between and bRSCs and bPCs with factors suspected to modulate henipaviruses infection from RNAi screening in the context of HeV infection [43]. This comparison highlighted among others NF-κB and cell death pathways. IL1R1 and CD40 membrane receptors involved in NFκB pathway were downregulated in bRSCs while upregulation of NF-κB regulator is observed in bRSCs such as NFKBIB (Figure 3f). RNA seq analysis reveals an intrinsic downregulation of TNFSF10 gene coding for TRAIL protein, FAS, CASP8, and CASP10 and upregulation of cell death inhibitor like BLC2 in batRSCs (Figure 3g). These factors are implicated in the modulation of cell death through necrosis, apoptosis, and autophagy. In summary, this ECM somatic reprogramming allows generating a new *Pteropus* bat cell type that expresses *Henipavirus* cellular entry receptor and presents a specific innate immune, inflammatory, and cell death gene signature.

### 3.7. bRSCs Are Highly Permissive for Henipavirus Infection with Low MOI

**Figure 4:**
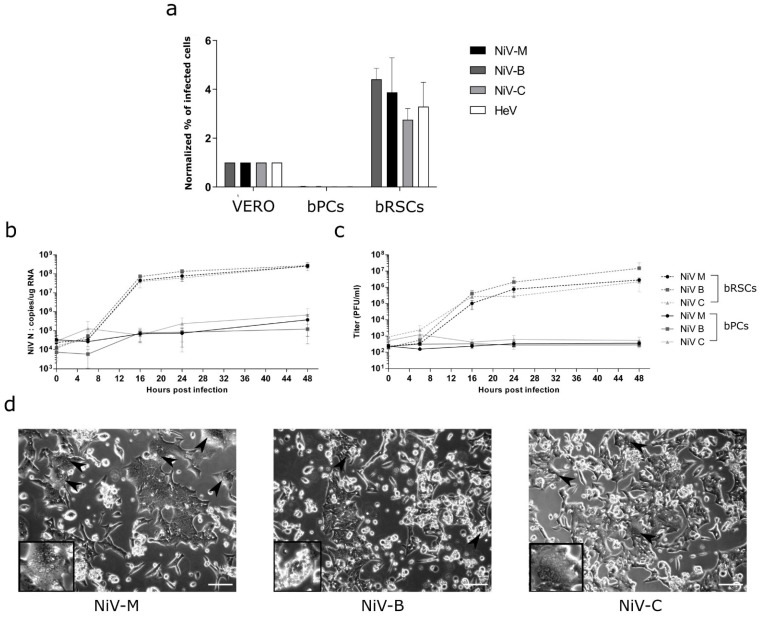
Susceptibility of bat reprogrammed cells (bRSCs) to henipaviruses. (**a**) Evaluation of entry of VSVΔG-RFP pseudotyped with *Henipavirus* glycoproteins into bPCs, bRSCs, and Vero cells. The infections of bPCs, bRSCs, and Vero cells were performed at an MOI of 0.01 and the percentages of infected cells were evaluated 6 h post-infection by measuring RFP by flow cytometry. (**b**–**d**) bPCs and bRSCs were infected with NiV-M, NiV-B and NiV-C isolate at an MOI of 0.1. The nucleocapsid gene transcription was quantified by RT-qPCR (**b**) and the release of virions into the supernatant was quantified by Vero plaque assay (**c**). (**d**) Cell cytopathic effects were observed under a light microscope at 24 h post-infection. Arrowheads show syncytia. Scale bar, 25 µm. Results labeled “bRSCs” correspond to bRSCs “EPI”. The same results were obtained with bRSCs “ESM2”.

We further tested the susceptibility of both bPCs and bRSCs to infection by the two human NiV isolates: NiV-M and NiV-B, as well as with one *Pteropus* bat NiV isolate: NiV-C and one equine Hendra virus isolate: HeV. bPCs, bRSCs, and Vero cells were infected with rVSVΔG-RFP viruses pseudotyped with henipaviruses glycoproteins at an MOI of 0.01. The percentage of infected cells was analyzed by flow cytometry and results from bPCs and bRSCs were normalized to the values obtained for Vero cells (Figure 4a). Similarly, the bRSCs have been also demonstrated to be more susceptible to Nipah virus infection than immortalized bPCs (Supplementary Figure S2). In contrast to bPCs, bRSCs were susceptible to infection with all four tested viruses. Henipaviruses entered bRSCs at a higher level than bPCs, in accord with the higher expression of the viral entry receptor ephrin B2 (Figure 3e). Interestingly, a higher level of entry was observed compared to Vero cells as well, which are commonly used to isolate and propagate henipaviruses. Next, we examined the transcription and replication kinetics of Nipah viruses by RT-qPCR and viral titration after infection of cells at a low MOI of 0.1 (Figure 4b,c). In bRSCs, NiV RNA synthesis and production of viral particles increased by 4 log10 units between 0 h and 24 h post-infection, while no increase was observed in bPCs. All of the tested viral isolates presented a similar level of viral replication, always at the higher level in bRSCs than in bPCs. Cytopathic effects and syncytia formation (arrowheads), hallmarks of NiV infection, were readily visible in bRSCs at 24 h post-infection (Figure 4d). We observed much more giant multinucleated cells in cultures infected with NiV-M and NiV-C compared to those with NiV-B. Surprisingly, despite the high level of infection and the cytopathic effects, a preserved bRSCs cell layer remained at 72 h post-infection unlike what is usually observed with Vero cell layers in a similar condition. Therefore, bRSCs are highly permissive to henipaviruses infection and are able to replicate and produce a high level of viruses.

## 4. Discussion

Efforts to combat deadly zoonotic infections caused by new emerging viruses are limited by a lack of knowledge about reservoir host biology and the absence of adequate in vitro models for studying host-pathogen interactions [13]. However, studying the virus in its natural host is crucial as different viruses have co-evolved with specific species. For example, the *Rousettus aegyptiacus* bat belonging to Pteropodidae fruit bats does not support productive NiV replication despite a conserved target for viral entry, which suggests that NiV replication is limited to only certain bat species [44]. Thus, the choice of a bat species for generating an in vitro model should be supported by virus susceptibility evidence. Many bat species are considered endangered; consequently, the sampling must be non-invasive or done during necropsies, which limits the diversity of the material available. The primary culture tends to select cells that grow easily in vitro like fibroblast cells, so working with these cells could limit our ability to study viruses like henipaviruses which present other cellular tropisms. In addition, bat cells have been poorly characterized so far, due to the lack of adequate bat-specific or cross-reactive reagents. By somatic reprogramming, we have aimed to derive and characterize new cells at both morphological and molecular levels.

In recent years, somatic reprogramming has become a powerful method for generating PSCs in species from which embryos or tissues are difficult to obtain. Unexpectedly, the OSKM reprogramming factors OCT4, SOX2, KLF4, and c-MYC, which are used successfully in other mammalian species [28], were inefficient in *Pteropus* cells, either alone or in combination with NANOG. This underlies the potential distinctiveness of bat cells with respect to this approach. Bats are described as unique, differing from other mammals in many aspects: metabolism, longevity, immunity, or DNA damage repair pathways. These bat features could affect somatic reprogramming as suggested in the naked mole rat cell reprogramming. Indeed, cells derived from naked mole rats, which present exceptional longevity and cancer resistance, resist somatic reprogramming with classical OSKM factor due to extreme stability of the epigenome [45]. In addition, most stem cell protocols have been designed for human and mouse cells; unconventional models may retain certain species-specific features that require thorough protocol optimization: choice of primary cell types, reprogramming strategies (combination, vectors), cell culture conditions (medium composition, coating, cell dissociation) [46]. Using the original combination of ESSRB, CDX2, and c-MYC transcription factors, we obtained bRSCs that exhibit stem cell-like properties. Future somatic reprogramming in other bat species will show whether the approach reported herein for *Pteropus* bat cells can be applied to other species as well. The reprogramming process presented in this report generated cells with rather unique stem cell characteristics. In addition to the expression of PSC-specific antigens and epigenetic markers, these reprogramed bat cells express an original transcriptomic signature including numerous neural stem cell markers, as evidenced by GO. As neural cells are one of the principal targets of henipaviruses, this cell profile may favor infection, as observed in our study. In addition, as is currently the case for human stem cells [47], further differentiation of bRSCs into neural cell types relevant for viral infection may open new ways for modeling viral infectious diseases.

Traditionally, either primary or immortalized cells have been used for virus infection studies in vitro. Primary cells retain their genetic integrity but are capable generally to undergo only a limited number of cell divisions before reaching senescence. bPCs generated in this study enter into senescence after 4 to 30 generations depending on the organ from which they originate. Our primary cultures resulted with only fibroblast-like cells, regardless of the medium used for the culture, and presented a very low and heterogeneous permissiveness to henipaviruses infection. Although immortalized cells are simple and cost-effective models suitable for genetic manipulation, they are very different from the primary cells from which they originate, especially in terms of cell signaling pathways, and they frequently accumulate chromosomal aberrations over time [12]. Moreover, those cells are also rarely characterized regarding the transcriptome and probably not highly permissive to infection by henipaviruses, as often the use of a high MOI during infection allows to detect of infected cells [23,25]. In contrast, the bRSC established in this study are highly infectable by henipaviruses, reaching 80 to 90% of infected cells. Comparison of gene expression, between refractory bPCs and susceptible bRSCs cells should allow the identification of factors controlling susceptibility or resistance to henipaviruses infections. These genes will be good candidates for functional studies by gain or loss of function.

Innate immunity is the first line of defense against viral infection. The antiviral response allows control of viral replication through several pathways such as viral recognition using pattern recognition receptors (PRRs), induction of interferon molecules (IFNs) and inflammatory cytokines, and apoptosis-related pathways. Bats innate immunity was suggested to present specific features compared to humans in many of these pathways [48,49]. The somatic reprogramming we performed with the ECM gene combination provides a new stem cell type with new properties in bat antiviral responses. Several factors related to the antiviral response are found to be differentially expressed between bPCs and bRSCs. First, among PRR, the downregulation in bRSCs of DDX58 and IFIH1 genes, coding for RIG-I and MDA5 respectively, previously shown to control NiV infection in a mouse model [50], suggests that bRSCs may be less able to detect and control henipaviruses infection. In addition, concerning IFNs and ISGs expression, neither bPCs nor bRSCs have a constitutive IFN gene expression, unlike what was previously seen in *P. Alecto* bats [42,51]. Surprisingly, the bRSCs present a specific intrinsic ISG profile with downregulation of 57 ISGs, indicating that ECM somatic reprogramming allows generating a new *Pteropus* bat cell type that presents a specific innate immune gene signature. This could explain why those cells are highly infectable compared to the bPCs. Moreover, factors involved in NFκB pathway are downregulated in bRSCs. The down-regulated pathways in highly susceptible bRSCs suggest that the factors associated could be important for the control of henipaviruses in bat cells. In our infection studies, we observed limited cell death in bRSCs despite a high level of infection. RNA seq analysis revealed an intrinsic downregulation of pro-apoptosis factors and an upregulation of cell death inhibitor in batRSCs. These factors are implicated in the modulation of cell death through necrosis, apoptosis, and autophagy, and those processes are known to be highly interconnected. Previous studies showed that henipaviruses induce apoptosis in bat cells but not in human cells and suggest that may contribute to controlling the viral infection in bats [52,53]. Moreover, ABLV infection of *P. Alecto* bat cells was characterized by less cell death in comparison to human cells, with an elevated basal autophagic level and autophagy process induced in response to high virus doses [54]. In a natural host, the cell death pathway is probably strongly modulated and responsible for allowing viral persistence at both cellular and tissue levels that could be the origin of the virus shedding without any disease symptoms.

Although the detection of virus-specific antibodies in bats and the use of molecular methods, such as viral sequencing, are often readily achievable, virus isolation from bats has been far more challenging. The isolation of henipaviruses has traditionally been tested in established immortalized cell lines, such as Vero cell lines which are deficient for the production of IFN-I. Isolation of henipaviruses in these cell lines can lead to the selection of viral metapopulations present in the host. The viruses isolated are the ones that replicate the most efficiently in Vero cells. In addition, this can lead to an accumulation of adaptive mutations, which have consequences for the study of host-pathogen interactions in vivo or in vitro [12]. The development of cell substrates from bats could allow the isolation of viral metapopulations from bats and also the production of viral stocks in the host, thus limiting viral drift during passages. The use of bRSCs, highly susceptible to henipaviruses infection may be a valuable tool for the isolation of new viruses and the production of viral stocks.

## 5. Conclusions

This study presents the generation of novel reprogrammed bat cells with stem cell-like features and high permissiveness for *Henipavirus* infection. Further analysis of the particular innate immune signature of these cells will help to improve our understanding of the mechanisms underlying bat antiviral responses and their utilisation may facilitate the isolation of novel bat Henipaviruses. This cell model should thus bridge an existing gap in this field, facilitate optimization of viral discovery in bats [55] and allow further research into bat biology at the cellular and molecular level. 

## 6. Patents

1. Method for reprogramming somatic cells (2018). Aurine N., Baquerre C., Horvat B., Pain B. EP18305321, WO2019180247 (A1)
2. Procédé de reprogrammation de cellules somatiques de ruminants. (2017). Jean C. Bacquerre C., Pain B. EP17305082

## Data Availability

The data sets are available in GSE134585 datasets.

## Supplementary Materials

The following are available online at https://www.mdpi.com/article/10.3390/microorganisms9122567/s1, Figure S1: Attempts to obtain *Pteropus* bRSCs using various delivery systems for reprogramming genes; Figure S2: *Pteropus medius* bat primary, immortalized by SV40 large-T as described [30] and reprogrammed cells were infected with the recombinant NiV-M virus expressing eGFP protein: rNiV-M-eGFP at a multiplicity of infection of 3. The percentage of infected cells was quantified at 24 h post-infection by measuring GFP expression by flow cytometry; Table S1: Reagents used for cell cultures; Table S2: List of genes expressed in either bPCs (PTC) or bRSCs in either EPI (ECM_E2) or ESM2 (ECM_ES2) media following RNAseq analysis as described in GSE134585 datasets; Table S3: List of ISG genes expressed in either bat PC or RSCs based on the consensual list of ISG described in other species and Table S4: Comparison of protein sequences of pluripotency genes between human and *Pteropus vampyrus* bat.

## References

[1] Jones K., Patel N., Levy M., Storeygard A., Balk D., Gittleman J.L., Daszak P. (2008). Global trends in emerging infectious diseases. Nat. Cell Biol..

[2] Luis A.D., Hayman D.T.S., O’Shea T.J., Cryan P., Gilbert A.T., Pulliam J., Mills J.N., Timonin M.E., Willis C., Cunningham A.A. (2013). A comparison of bats and rodents as reservoirs of zoonotic viruses: Are bats special?. Proc. R. Soc. B Boil. Sci..

[3] Olival K.J., Hosseini P.R., Zambrana-Torrelio C., Ross N., Bogich T., Daszak P. (2017). Host and viral traits predict zoonotic spillover from mammals. Nat. Cell Biol..

[4] Halpin K., Daszak P., Field H.E., Hyatt A.D., Smith C., Epstein J.H., Middleton D., Fogarty R., Hughes T., Bingham J. (2011). Pteropid Bats are Confirmed as the Reservoir Hosts of Henipaviruses: A Comprehensive Experimental Study of Virus Transmission. Am. J. Trop. Med. Hyg..

[5] Wang L.-F., Anderson D.E. (2019). Viruses in bats and potential spillover to animals and humans. Curr. Opin. Virol..

[6] Leroy E.M., Kumulungui B., Pourrut X., Rouquet P., Hassanin A., Yaba P., Délicat A., Paweska J.T., Gonzalez J.-P., Swanepoel R. (2005). Fruit bats as reservoirs of Ebola virus. Nature.

[7] Banyard A.C., Hayman D., Johnson N., McElhinney L., Fooks A.R. (2011). Bats and Lyssaviruses. Adv. Virus Res..

[8] Li W., Shi Z., Yu M., Ren W., Smith C., Epstein J.H., Wang H., Crameri G., Hu Z., Zhang H. (2005). Bats Are Natural Reservoirs of SARS-Like Coronaviruses. Science.

[9] Mohd H.A., Al-Tawfiq J.A., Memish Z.A. (2016). Middle East Respiratory Syndrome Coronavirus (MERS-CoV) origin and animal reservoir. Virol. J..

[10] El-Sayed A., Kamel M. (2021). Coronaviruses in humans and animals: The role of bats in viral evolution. Environ. Sci. Pollut. Res..

[11] Enchéry F., Horvat B. (2017). Understanding the interaction between henipaviruses and their natural host, fruit bats: Paving the way toward control of highly lethal infection in humans. Int. Rev. Immunol..

[12] Banerjee A., Misra V., Schountz T., Baker M.L. (2018). Tools to study pathogen-host interactions in bats. Virus Res..

[13] Hare D., Collins S., Cuddington B., Mossman K. (2016). The Importance of Physiologically Relevant Cell Lines for Studying Virus–Host Interactions. Viruses.

[14] Mathieu C., Horvat B. (2014). Henipavirus pathogenesis and antiviral approaches. Expert Rev. Anti-Infect. Ther..

[15] Fouret J., Brunet F.G., Binet M., Aurine N., Enchéry F., Croze S., Guinier M., Goumaidi A., Preininger D., Volff J.-N. (2020). Sequencing the Genome of Indian Flying Fox, Natural Reservoir of Nipah Virus, Using Hybrid Assembly and Conservative Secondary Scaffolding. Front. Microbiol..

[16] Middleton D., Westbury H., Morrissy C., van der Heide B., Russell G., Braun M., Hyatt A. (2002). Experimental Nipah Virus Infection in Pigs and Cats. J. Comp. Pathol..

[17] Ching P.K.G., Reyes V.C.D.L., Sucaldito M.N., Tayag E., Columna-Vingno A.B., Malbas F.F., Bolo G.C., Sejvar J.J., Eagles D., Playford G. (2015). Outbreak of Henipavirus Infection, Philippines, 2014. Emerg. Infect. Dis..

[18] Luby S.P., Rahman M., Hossain M.J., Blum L.S., Husain M.M., Gurley E., Khan R., Ahmed B.-N., Rahman S., Nahar N. (2006). Foodborne Transmission of Nipah Virus, Bangladesh. Emerg. Infect. Dis..

[19] Nikolay B., Salje H., Hossain M.J., Khan A.D., Sazzad H., Rahman M., Daszak P., Ströher U., Pulliam J.R., Kilpatrick A.M. (2019). Transmission of Nipah Virus — 14 Years of Investigations in Bangladesh. New Engl. J. Med..

[20] Wong K.T., Shieh W.-J., Kumar S., Norain K., Abdullah W., Guarner J., Goldsmith C.S., Chua K.B., Lam S.K., Tan C.T. (2002). Nipah Virus Infection. Am. J. Pathol..

[21] Drexler J.F., Corman V.M., Müller M.A., Maganga G.D., Vallo P., Binger T., Gloza-Rausch F., Cottontail V.M., Rasche A., Yordanov S. (2012). Bats host major mammalian paramyxoviruses. Nat. Commun..

[22] Luby S.P. (2013). The pandemic potential of Nipah virus. Antivir. Res..

[23] Crameri G., Todd S., Grimley S., McEachern J.A., Marsh G.A., Smith C., Tachedjian M., De Jong C., Virtue E.R., Yu M. (2009). Establishment, Immortalisation and Characterisation of Pteropid Bat Cell Lines. PLoS ONE.

[24] Glennon N.B., Jabado O., Lo M.K., Shaw M.L. (2015). Transcriptome Profiling of the Virus-Induced Innate Immune Response in Pteropus vampyrus and Its Attenuation by Nipah Virus Interferon Antagonist Functions. J. Virol..

[25] Virtue E.R., Marsh G.A., Baker M.L., Wang L.-F. (2011). Interferon Production and Signaling Pathways Are Antagonized during Henipavirus Infection of Fruit Bat Cell Lines. PLOS ONE.

[26] Hoffmann M., Müller M.A., Drexler J.F., Glende J., Erdt M., Gützkow T., Losemann C., Binger T., Deng H., Schwegmann-Weßels C. (2013). Differential Sensitivity of Bat Cells to Infection by Enveloped RNA Viruses: Coronaviruses, Paramyxoviruses, Filoviruses, and Influenza Viruses. PLoS ONE.

[27] Adachi K., Kopp W., Wu G., Heising S., Greber B., Stehling M., Araúzo-Bravo M.J., Boerno S.T., Timmermann B., Vingron M. (2018). Esrrb Unlocks Silenced Enhancers for Reprogramming to Naive Pluripotency. Cell Stem Cell.

[28] Rayon T., Menchero S., Rollán I., Ors I., Helness A., Crespo M., Nieto A., Azuara V., Rossant J., Manzanares M. (2016). Distinct mechanisms regulate Cdx2 expression in the blastocyst and in trophoblast stem cells. Sci. Rep..

[29] Takahashi K., Yamanaka S. (2006). Induction of Pluripotent Stem Cells from Mouse Embryonic and Adult Fibroblast Cultures by Defined Factors. Cell.

[30] Gaudino M., Aurine N., Dumont C., Fouret J., Ferren M., Mathieu C., Reynard O., Volchkov V.E., Legras-Lachuer C., Georges-Courbot M.-C. (2020). High Pathogenicity of Nipah Virus fromPteropus lyleiFruit Bats, Cambodia. Emerg. Infect. Dis..

[31] Aubel P., Pain B. (2013). Chicken Embryonic Stem Cells: Establishment and Characterization. Adv. Struct. Saf. Stud..

[32] Coronado D., Godet M., Bourillot P.-Y., Tapponnier Y., Bernat A., Petit M., Afanassieff M., Markossian S., Malashicheva A., Iacone R. (2013). A short G1 phase is an intrinsic determinant of naïve embryonic stem cell pluripotency. Stem Cell Res..

[33] Kress C., Montillet G., Jean C., Fuet A., Pain B. (2016). Chicken embryonic stem cells and primordial germ cells display different heterochromatic histone marks than their mammalian counterparts. Epigenetics Chromatin.

[34] Wu X., Thi V.L.D., Huang Y., Billerbeck E., Saha D., Hoffmann H.-H., Wang Y., Silva L.A.V., Sarbanes S., Sun T. (2018). Intrinsic Immunity Shapes Viral Resistance of Stem Cells. Cell.

[35] Yoneda M., Guillaume V., Ikeda F., Sakuma Y., Sato H., Wild T.F., Kai C. (2006). Establishment of a Nipah virus rescue system. Proc. Natl. Acad. Sci. USA.

[36] Mathieu C., Guillaume V., Sabine A., Ong K.C., Wong K.T., Legras-Lachuer C., Horvat B. (2012). Lethal Nipah Virus Infection Induces Rapid Overexpression of CXCL10. PLoS ONE.

[37] Fuet A., Montillet G., Jean C., Aubel P., Kress C., Rival-Gervier S., Pain B. (2018). NANOG is Required for the Long-Term Establishment of Avian Somatic Reprogrammed Cells. Stem Cell Rep..

[38] Trusler O., Huang Z., Goodwin J., Laslett A.L. (2018). Cell surface markers for the identification and study of human naive pluripotent stem cells. Stem Cell Res..

[39] Zalzman M., Falco G., Sharova L.V., Nishiyama A., Thomas M., Lee S.-L., Stagg C.A., Hoang H.G., Yang H.-T., Indig F.E. (2010). Zscan4 regulates telomere elongation and genomic stability in ES cells. Nat. Cell Biol..

[40] Bossart K.N., Tachedjian M., McEachern J.A., Crameri G., Zhu Z., Dimitrov D.S., Broder C.C., Wang L.-F. (2008). Functional studies of host-specific ephrin-B ligands as Henipavirus receptors. Virology.

[41] Negrete O.A., Levroney E.L., Aguilar H.C., Bertolotti-Ciarlet A., Nazarian R., Tajyar S., Lee B. (2005). EphrinB2 is the entry receptor for Nipah virus, an emergent deadly paramyxovirus. Nat. Cell Biol..

[42] Woon A.P., Boyd V., Todd S., Smith I., Klein R., Woodhouse I.B., Riddell S., Crameri G., Bingham J., Wang L.-F. (2020). Acute experimental infection of bats and ferrets with Hendra virus: Insights into the early host response of the reservoir host and susceptible model species. PLoS Pathog..

[43] Deffrasnes C., Marsh G.A., Foo C.H., Rootes C.L., Gould C.M., Grusovin J., Monaghan P., Lo M., Tompkins S.M., Adams T. (2016). Genome-wide siRNA Screening at Biosafety Level 4 Reveals a Crucial Role for Fibrillarin in Henipavirus Infection. PLoS Pathog..

[44] Seifert S., Letko M.C., Bushmaker T., Laing E.D., Saturday G., Meade-White K., Van Doremalen N., Broder C.C., Munster V.J. (2019). Rousettus aegyptiacus Bats Do Not Support Productive Nipah Virus Replication. J. Infect. Dis..

[45] Tan L., Ke Z., Tombline G., Macoretta N., Hayes K., Tian X., Lv R., Ablaeva J., Gilbert M., Bhanu N.V. (2017). Naked Mole Rat Cells Have a Stable Epigenome that Resists iPSC Reprogramming. Stem Cell Rep..

[46] Ou J., Rosa S., Berchowitz L.E., Li W. (2019). Induced pluripotent stem cells as a tool for comparative physiology: Lessons from the thirteen-lined ground squirrel. J. Exp. Biol..

[47] Trevisan M., Sinigaglia A., Desole G., Berto A., Pacenti M., Palù G., Barzon L. (2015). Modeling Viral Infectious Diseases and Development of Antiviral Therapies Using Human Induced Pluripotent Stem Cell-Derived Systems. Viruses.

[48] Mandl J.N., Schneider C., Schneider D.S., Baker M. (2018). Going to Bat(s) for Studies of Disease Tolerance. Front. Immunol..

[49] Banerjee A., Baker M.L., Kulcsar K., Misra V., Plowright R., Mossman K. (2020). Novel Insights Into Immune Systems of Bats. Front. Immunol..

[50] Iampietro M., Aurine N., Dhondt K.P., Dumont C., Pelissier R., Spanier J., Vallve A., Raoul H., Kalinke U., Horvat B. (2019). Control of Nipah Virus Infection in Mice by the Host Adaptors Mitochondrial Antiviral Signaling Protein (MAVS) and Myeloid Differentiation Primary Response 88 (MyD88). J. Infect. Dis..

[51] Zhou P., Tachedjian M., Wynne J., Boyd V., Cui J., Smith I., Cowled C., Ng J.H.J., Mok L., Michalski W.P. (2016). Contraction of the type I IFN locus and unusual constitutive expression ofIFN-αin bats. Proc. Natl. Acad. Sci. USA.

[52] Wynne J.W., Shiell B.J., A Marsh G., Boyd V., A Harper J., Heesom K., Monaghan P., Zhou P., Payne J., Klein R. (2014). Proteomics informed by transcriptomics reveals Hendra virus sensitizes bat cells to TRAIL mediated apoptosis. Genome Biol..

[53] Chen M., Tachedjian M., Marsh G.A., Cui J., Wang L.-F. (2020). Distinct Cell Transcriptomic Landscapes Upon Henipavirus Infections. Front. Microbiol..

[54] Laing E.D., Sterling S.L., Weir D.L., Beauregard C.R., Smith I.L., Larsen S.E., Wang L.-F., Snow A.L., Schaefer B.C., Broder C.C. (2019). Enhanced Autophagy Contributes to Reduced Viral Infection in Black Flying Fox Cells. Viruses.

[55] Young C.C.W., Olival K.J. (2016). Optimizing Viral Discovery in Bats. PLoS ONE.

