# Supplementary material for: Reprogrammed Pteropus Bat Stem Cells as A Model to Study Host-Pathogen Interaction during Henipavirus Infection
Source: Microorganisms. 2021 Dec 11;9(12):2567. doi: 10.3390/microorganisms9122567 (PMC8706405; doi:10.3390/microorganisms9122567)
Supplement: Supplementary file 1 [file microorganisms-09-02567-s001.zip › microorganisms-1460759-Supplementary Table S1, S4 and Figure S1.pdf]

microorganisms

MDPI

Article

Reprogrammed *Pteropus* Bat Stem Cells as A Model to Study Host-Pathogen Interaction During *Henipavirus* Infection

Noémie Aurine [1,2], Camille Baquerre [1], Maria Gaudino [2], Christian Jean [1], Claire Dumont [2], Sylvie Rival-Gervier [1], Clémence Kress [1], Branka Horvat [2,*] and Bertrand Pain [1,*]

1 Stem Cell and Brain Research Institute, University of Lyon, Université Lyon 1, INSERM, INRAE, U1208, USC1361, Bron, France; noemie.aurine@inserm.fr (N.A.); camille.baquerre@inserm.fr (C.B.); christian.jean@inserm.fr (C.J.); sylvie.gervier@inserm.fr (S.R.-G.); clemence.kress@inserm.fr (C.K.)
2 CIRI, International Center for Infectiology Research, University of Lyon, Université Claude Bernard Lyon 1, INSERM, U1111, CNRS, UMR5308, ENS Lyon, Lyon, France; mariagaudino01@gmail.com (M.G.); dumont.claire3@gmail.com (C.D.)
* Correspondence: bertrand.pain@inserm.fr (B.P.); branka.horvat@inserm.fr (B.H.); Tel.: +33-437282392

Supplementary Material

Table S1. Reagents used for cell cultures.

Reagent	Provider	Reference	Batch
DMEM/F-12, no glutamine	Thermo Fisher Scientific	21331020	1838085
Fetal Bovine Serum, qualified, E.U.-approved, South America origin	Thermo Fisher Scientific	10270106	42Q3563K
Penicillin-Streptomycin (10,000 U/mL)	Thermo Fisher Scientific	15140122	1864853
L-Glutamine (200 mM)	Thermo Fisher Scientific	25030024	1839916
MEM Non-Essential Amino Acids Solution (100X)	Thermo Fisher Scientific	11140050	17A76622
Sodium Pyruvate (100 mM)	Thermo Fisher Scientific	11360070	1815685
2-Mercaptoethanol	Thermo Fisher Scientific	31350-010	1815203
IL-6	Peprotech	200-06	031316
IL6 receptor	Peprotech	200-06R	135243
Mouse Stem Cell Factor (mSCF)	Peprotech	300-07	091578
insulin-like growth factor-1 (IGF1)	Peprotech	100-11	101401-2
LIF			
Neurobasal™ Medium	Thermo Fisher Scientific	21103049	1801691
B-27 Supplement (50X), serum free	Thermo Fisher Scientific	17504044	1860074
N-2 Supplement (100X)	Thermo Fisher Scientific	17502048	1865578
Recombinant Human FGF-basic (154 a.a.)	Peprotech	100-18B	071608 I1416
Recombinant Human/Murine/Rat Activin A (E. coli derived)	Peprotech	120-14E	0712478 B0915
Doxycycline hyclate	SIGMA	D981	BCBP0625V

Table S2. List of genes expressed in either bPCs (PTC) or bRSCs in either EPI (ECM_E2) or ESM2 (ECM_ES2) media following RNAseq analysis as described in GSE134585 datasets. Gene ID number (geneid) and Gene symbol (symbol) were provided as well as the Log of fold change (LFC) of both bRSCs compared to PTC.

Available in additional file

Table S3. List of ISG genes expressed in either bat PC or RSCs based on the consensual list of ISG described in other species and used for the heatmap clustering. Values were extracted from the complete list as mentioned in Table S3.

Available in additional file

Table S4. Comparison of protein sequences of pluripotency genes between human and *Pteropus vampyrus* bat.

Gene	*Homo Sapiens* Protein ID	*Pteropus vampyrus* Protein ID	% Alignment (Proteins) Between *Homo Sapiens* and *Pteropus vampyrus* Species
OCT4	NP_002692.2	XP_011382211.1	90.9
SOX2	NP_003097.1	XP_011381167.1	99.1
KLF4	NP_001300981.1	XP_011363252.1	87.3
C-MYC	NP_002458.2	XP_011359121.1	92.0
NANOG	NP_079141.2	XP_011364079.1	69.9
CDX2	NP_001256.3	XP_011358539.1	91.0
ESRRB	NP_004443.3	XP_011355347.1	98.8

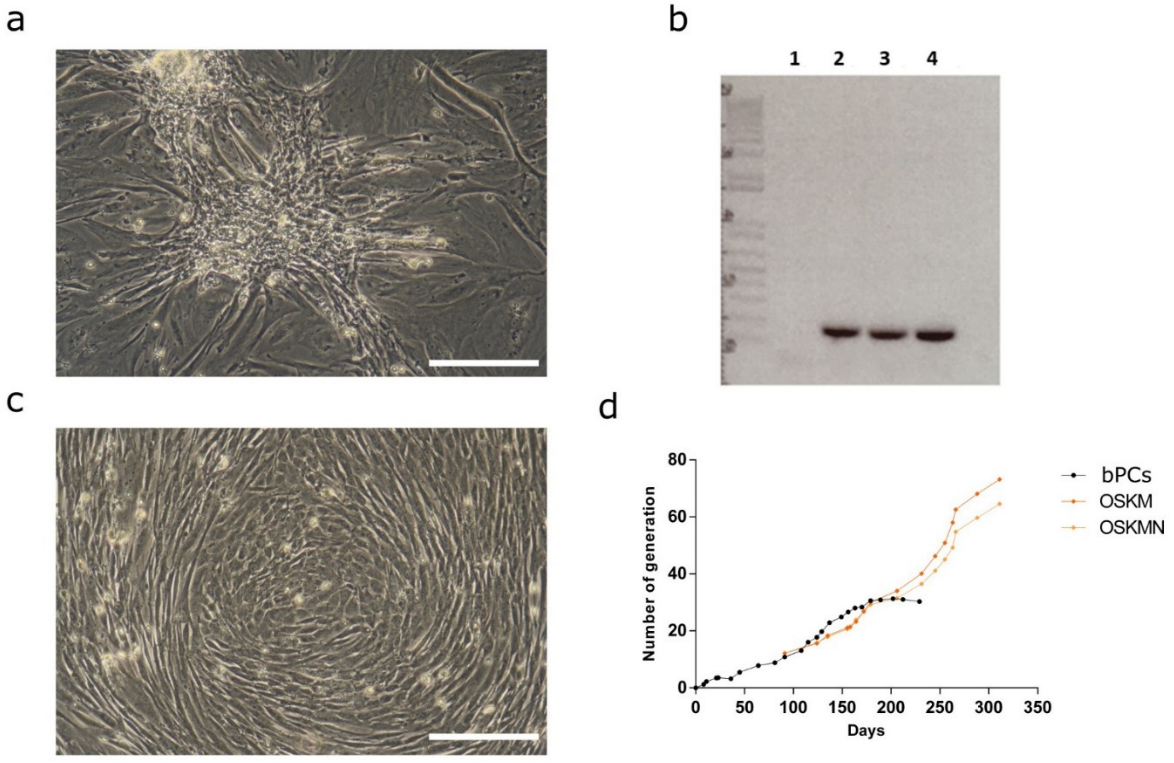

Figure S1. Attempts to obtain *Pteropus* bRSCs using various delivery systems for reprogramming genes. (**a**) The classical OSKM combination was introduced into bat primary cells (bPCs) using a non-integrative Sendai virus and cells were observed under a light microscope (scale bar, 200 µm). (**b**) PCR-based detection of Sendai virus was performed as recommended by the supplier: 1, bPCs; 2, bPCs + Sendai; 3, bPCs + Sendai + NANOG; 4, Control reprogrammed stem cells. (**c**) bPCs were modified by electroporation of inducible transposons encoding OCT4, SOX2, KLF4, and c-MYC. Scale bar, 200 µm. (**d**) Comparative growth curves of bPCs modified by electroporation of OSKM and OSKMN.
