# Supplementary figures and images for: Reprogrammed Pteropus Bat Stem Cells as A Model to Study Host-Pathogen Interaction during Henipavirus Infection
Source: Microorganisms. 2021 Dec 11;9(12):2567. doi: 10.3390/microorganisms9122567 (PMC8706405; doi:10.3390/microorganisms9122567)

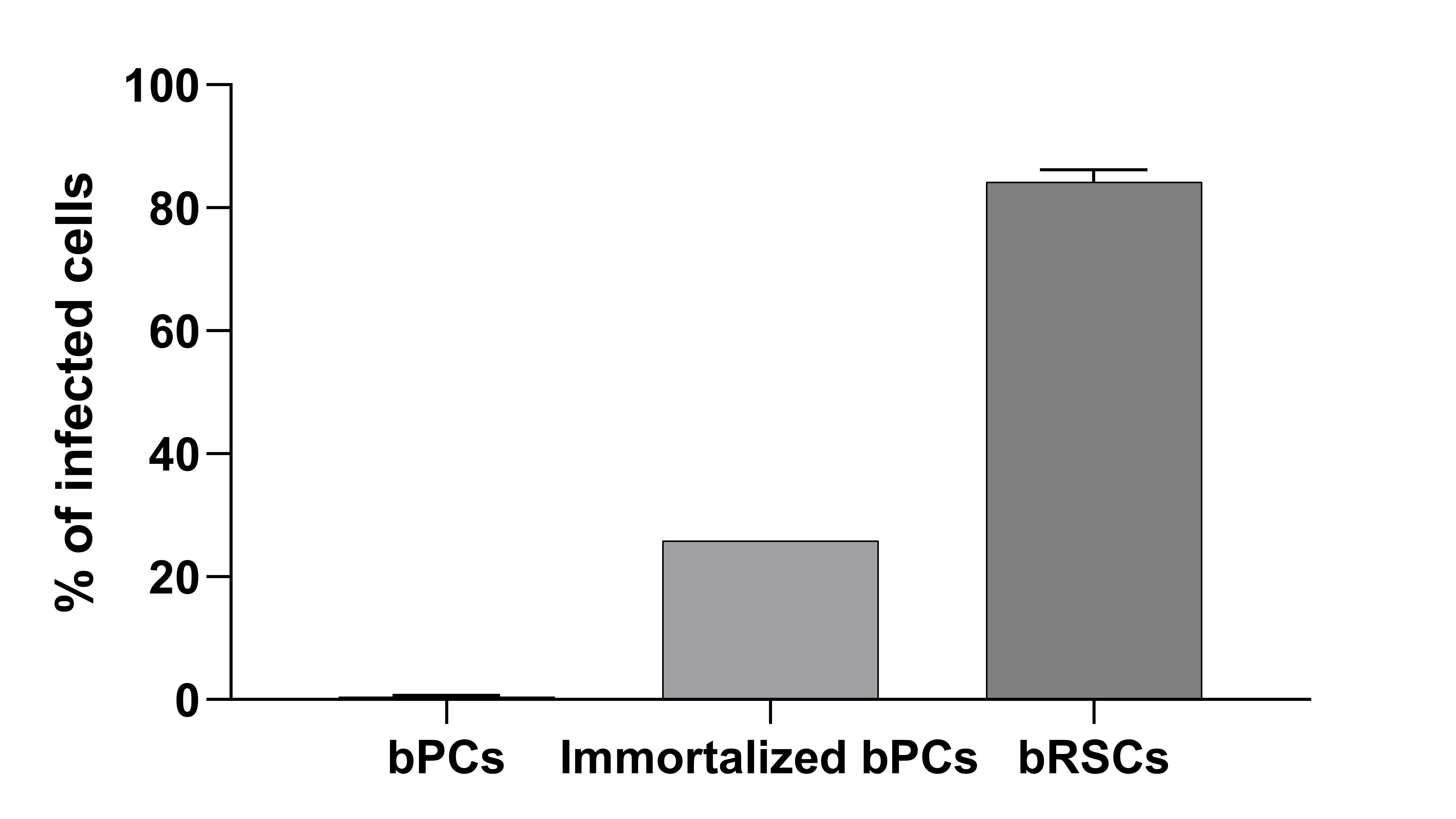

Supplement: Supplementary file 1 [file microorganisms-09-02567-s001.zip › microorganisms-1460759-supplementary-Figure S2.jpg]
